# Cymene and Metformin treatment effect on biochemical parameters of male NMRI mice fed with high fat diet

**DOI:** 10.1186/s40200-015-0182-x

**Published:** 2015-06-24

**Authors:** Peyman Lotfi, Parichehreh Yaghmaei, Azadeh Ebrahim-Habibi

**Affiliations:** Department of Biology, Science and Research Branch, Islamic Azad University, Tehran, Iran; Biosensor Research Center, Endocrinology and Metabolism Molecular-Cellular Sciences Institute, Tehran University of Medical Sciences, Shariati Hospital, North Kargar Avenue, 1411413137 Tehran, Iran; Endocrinology and Metabolism Research Center, Endocrinology and Metabolism Clinical Sciences Institute, Tehran University of Medical Sciences, Tehran, Iran

**Keywords:** Obesity, *p*-cymene, Metformin, NMRI mice

## Abstract

**Background:**

The increasing prevalence of obesity is considered a serious global health threat. Mainly due to change of diet and reduced physical activity, obesity is an important risk factor for chronic diseases. A higher level of cytokines and a general inflammatory state has also been associated with this condition. With this regard, potential anti-obesity compounds with anti-inflammatory properties could be beneficial in better control of the disease. *p*-Cymene is a natural aromatic compound that has been shown to have anti-inflammatory properties, while the antidiabetic drug metformin has been observed to be effective as an aid for weight loss. In this study, the effect of these comounds was compared in a high fat diet treated mice model.

**Methods:**

48 adult NMRI mice were randomly divided into six groups: control group receiving a normal diet, high fat diet (HFD) fed control group, sham group receiving HFD and sunflower seed oil, Experimental group1 (E1) receiving HFD and 20 mg/kg metformin, Experimental group2 (E2) receiving 20 mg/kg metformin and 20 mg/kg *p*-cymene, Experimental group3 (E3) receiving 20 mg/kg *p*-cymene. Compounds were administered by intragastric gavage for 45 days.

**Results:**

Non-fasting glucose serum levels, ALT, and ALP of E2 and E3 decreased significantly compared to HFD control group. In the E3 group, AST levels decrease was also significant. In E1, non-fasting glucose and TG serum levels decreased significantly compared to HFD control group. Histological observations on liver tissue showed an increase of lipid droplets in the HFD control group compared with the normal group, while upon treatment with the compounds, lipid droplets decreased and the cells appeared to be more ordered.

**Conclusion:**

*p*-Cymene has a potential to ameliorate biochemical parameters in high fat diet treated mice, and its concurrent use with metformin was effective.

## Background

The global growing prevalence of obesity is usually attributed to changes in the life style of modern societies, including the important factor of high-fat diets consumption [1, 2]. The overall increase of fat intake, and the higher energy content of fats (9 kcal per gram compared with 4 kcal per gram for carbohydrates) are both important in the development of obesity [3], but genetic factors should also be taken into account [4]. Obesity predisposes to a variety of metabolic diseases, and is related with metabolic syndrome, which is characterized by factors such as central obesity, insulin resistance, increased arterial pressure, and non-alcoholic steatohepatitis (NASH) [5, 6]. Moreover, metabolic syndrome increases the risk of type 2 diabetes and cardiovascular diseases [7]. Epidemiological studies show that nutritional habits such as a high consumption of saturated fat and cholesterol can influence the prevalence of metabolic syndrome [8].

Obesity is associated with a chronic inflammatory response, where abnormal adipokine production and activation of pro-inflammatory signaling pathways would result into induction of several biological markers of inflammation [9]. Accordingly, a reduction in body weight is followed by a decrease or even a normalization of these parameters [10, 11]. Reports on animal models support the existence of a link between inflammatory processes and obesity, as well as it co-morbidities [12, 13]. In addition to their role in metabolic disorders, fat cells potential role in inflammatory process is relatively recently introduced. Moreover, several findings point to common characteristics between inflammatory and immune cells such as complement activation and pro-inflammatory cytokine production [14]. Similar to macrophages, preadipocytes have the capacity for phagocytosis and have been demonstrated to resemble more to macrophages than adipocytes [15, 16]. These findings are suggestive of the possibility for compounds with anti-inflammatory properties to become potential anti-obesity candidates.

*p*-Cymene (p-isopropyltoluene) is a natural aromatic organic compound [17] used as a flavoring agent and in the production of chemicals [18]. It is an important component of plants belonging to the *Thymus* genus, has a simple structure similar to thymol, and is the biological precursor of carvacrol [19]. As part of volatile oils extracted from plants, cymene has been reported to have anti-bacterial and anti-oxidant properties [19, 20]. Recent studies have demonstrated interesting analgesic and anti-inflammatory effect of *p*-cymene itself [21–23]. With regard to these properties, we aimed at investigating the effect of cymene on mice fed with high-fat diet, which could be considered to be at risk of developing obesity. Cymene effect was compared with metformin. Metformin is a safe glucose-lowering drug which has some other indications such as treating polycystic ovarian syndrome [24], aiding in weight loss [25], and some cases of impaired glucose tolerance [26].

## Methods

### Animals

48 adult male NMRI mice weighing approximately 20–25 g were housed under standard laboratory conditions with a 12 h light–dark cycle. Animals were and fed with rodent pellets and tap water for 1 week before starting the experiments. Animal welfare and experimental procedures were carried out strictly in accordance with the Guide for the Care and Use of Laboratory Animals [27], and approval had been received from the Islamic Azad University, Science and Research Branch Animal Ethics Committee prior to making the experiments.

### Experimental design

After accommodation for one week, mice were randomly divided into six groups of eight animals as follows: (1) the normal group that received standard rodent diet without any food restriction; (2) high fat diet control group that received a high fat diet (HFD); (3) sham group that received drug solvent (sunflower seed oil) alongside with HFD; (4) Experimental group1 that received Fat diet plus 20 mg / kg metformin; (5) Experimental group 2 that received HFD plus 20 mg/kg cymene; (6) Experimental group 3 that received HFD with both metformin and cymen at doses of 20 mg/kg. The experiment duration was 6 weeks and compounds were given by oral gavage.

HFD was prepared from a mix of 15 g of mouse pellet standard chow, 10 g of roasted ground nut, 10 g of milk chocolate and 5 g of sesame crackers; 20 g roasted sesame was added to ten-fold of these components, which amounts into 18 kJ/g. The high fat diet treated group was also given 240 g creamy biscuits weekly [28].

### Chemicals

*p*-Cymene (99.7 % purity) was purchased from Sigma, USA. Metformin was from Kimi Daru Pharmaceutical Co, Iran. Commercial kits used for determination of blood glucose, cholesterol, triglyceride (TG), HDL-C,LDL-C, alanine aminotransferase (ALT), and alkaline phosphatase (ALP) were from Pars Azmoon Company, Iran.

### Measured parameters

At the end of the experiment, body weights of animals were measured; animals were anesthetized by inhalation of mild diethyl ether, and their blood was taken. The blood samples were allowed to clot for 30 min at room temperature and centrifuged at 1000 × g at 37 °C for 10 min to separate the serum. Levels of non-fasting glucose, cholesterol, TG, HDL-C,LDL-C and the activities of ALT, AST and ALP were determined.

### Statistical analysis

Results are expressed as mean ± S.E.M. Statistical analysis was performed using one-way ANOVA test followed by a Tukey posthoc test. *p* < 0.05 was considered statistically significant.

## Results

### Body weight

After the end of the experiment, group 1–6 weights were measured. As shown in Table 1, no significant difference was observed in this regard between different groups.

**Table 1:** Effects of Metformin and Cymene treatment on Body Weight(g),serum non-fasting Glucose, Cholesterol (Chol), triglyceride (TG), ALT,ALP of the mice at the week6

	Control (1)	High fat diet (2)	Sham(3)	Metformin (4)	Cymene (5)	Metformin + Cymene (6)
Weight(gr)	41 ±.86	40.50±.76	41.5±1.04	39.5±.1.04	40±1.15	40.071±.80
Non-fasting Glucose	273±10.4	*320±7.64	*325±11.06	++261±4.58	+++243±7.02	+273±9.7
Chol	135±.4.5	150±.11	152±11.3	139±3.7	144±5.5	146±9
TG	120±3.6	136±.7.02	147±8.54	+96±7.63	129±8.08	110±7.02
ALT	54±3.71	67±4.04	60±3.05	53±3.06	+++43±3.05	+++38±2.51
ALP	201±5.85	220±5.29	210±7.21	200±8.54	++172±9.165	*179±10

ALP.Data are shown as mean ± SEM. *p < 0.05 compared with the normal control group, +++*p* < 0.001 compared with the high fat diet group, ++*p* < 0.01 compared with the high fat diet group, +*p* < 0.05 compared with the high fat diet group

### Non-fasting blood glucose

Non-fasting Blood glucose increased significantly upon taking HFD as well as in sham groups, compared to the control group (*p* < 0.05).

Treatment with *p*-cymene alone, metformin alone and metformin/cymene complex resulted in significantly decreased levels of blood glucose as compared with the control group, with (*p* < 0.001), (*p* < 0.01) and (*p* < 0.05) respectively (Table 1).

### Lipids

Cholesterol levels of the untreated group taking HFD, as well as the sham one were higher than the control group (although not significanltly) (Table 1). Between treated groups, giving metformin alone had a better lowering effect on cholesterol (but not significantly). TG levels, which were increased to some extent upon consumption of HFD as well as in the sham group, were significantly decreased upon metformin treatment (*p* < 0.05), while other treatments, although effective, could not significantly influence TG levels (Table 1).

### ALT and ALP

Serum ALT and ALP levels of HFD-given and sham groups were higher than those in the control group. Upon both treatments, a significant lowering of ALT levels occurred compared with the HFD group (*p* < 0.001), while treatment with the cymene and metformin combination made a significant difference with the normal control group (*p* < 0.05). ALP levels were also significantly affected by the combinative treatment (*p* < 0.05) and cymene treatment (*p* < 0.01) compared with the HFD group. Metformine alone had no significant effect in this regard.

### Histological analysis

**Fig. 1:**
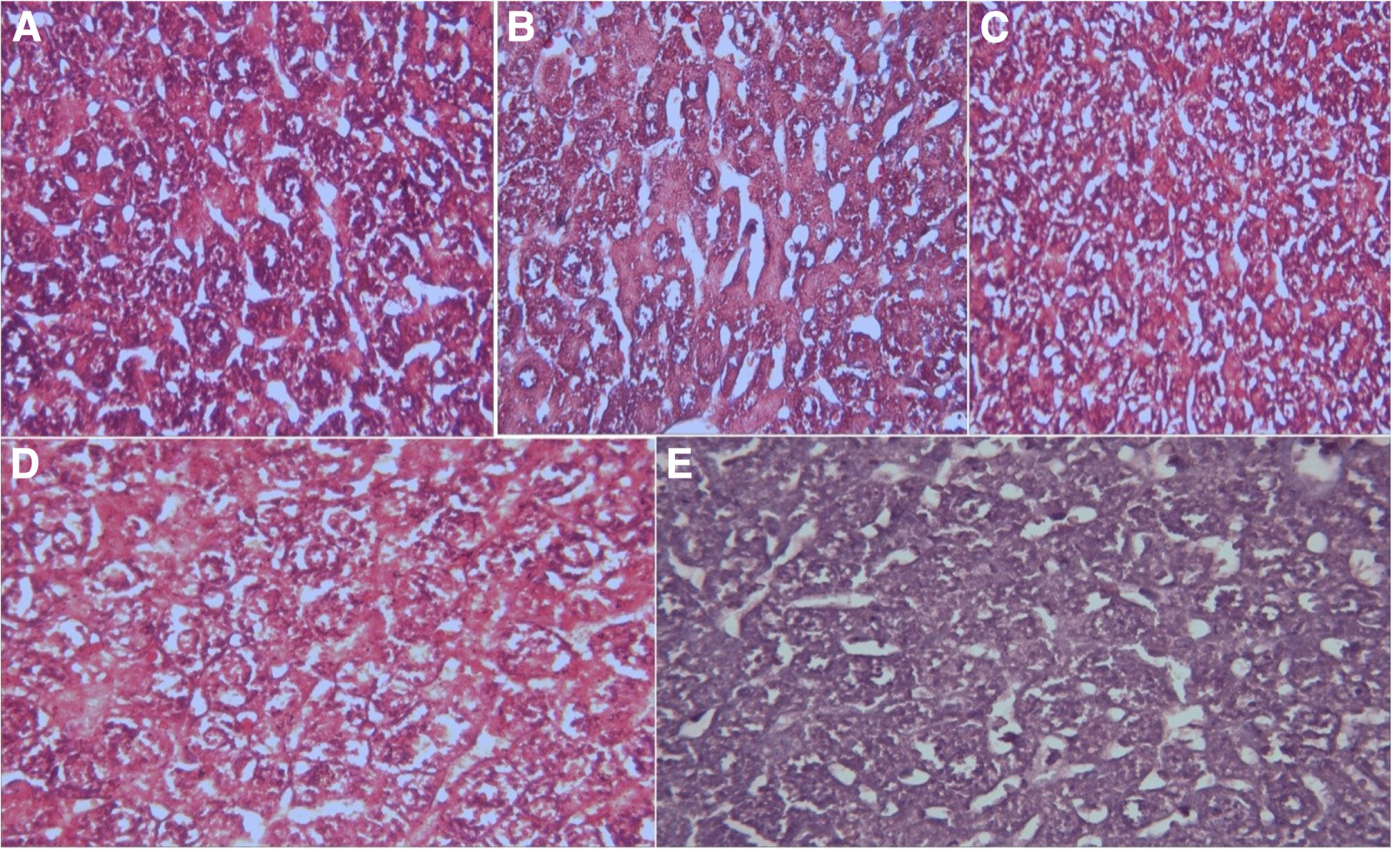
Trichrome-stained sections of liver from control (**a**), HFD-given (**b**), metformin-treated (**c**), metformin + p-Cymene (**d**), and *p*-cymene (**e**) groups. Magnification is of (400×)

Histological examination with trichrome staining of liver sections from HFD-fed mice demonstrated the development of steatosis as shown in Fig. 1. The fat droplets were decreased in the groups that had received either metformin or metformin combination with cymene.

## Discussion

In the present study, the body weight of animals did not change significantly upon treatment with metformin. Typical weight loss reported upon use of metformin is modest [29]. As an example, a weight loss of 3 kg over 16 weeks with metformin and a weight-maintaining diet may be cited [30]. The present study period is also a limitation in this regard. Interestingly, thymol, which is has a similar structure to *p*-cymene has been reported to decrease animals weight which had been treated with a high-fat diet [31].

On the other hand, metformin treatment resulted into significant decrease of blood glucose levels of HFD-treated animals (around 20 %). Metformin is reported to suppress glucose production in the liver and may result to 25–30 % reduction of fasting plasma glucose concentrations [30, 32]. *P-*cymene and its combination with metformin were also effective in this regard, a fact that was observed with thymol too [31]. In this regard, *p*-cymene could have a potentially positive effect in diabetic state, which should be checked in suitable diabetic models.

Lipid profile was influenced by the treatment, although overall not significantly. The only significant effect was obtained by the use of metformin on TG levels. Meta-analyses have shown positive although small effect of metformin on lipid profile factors in diabetic patients [33], while combination of metformin with other noninsulin anti-hyperglycemic drugs was found to be moderate to small [34]. In NAFLD patients, as well as obese non-diabetic patients various results had been obtained, with effectiveness of metformin shown in some cases [35, 36]. In other reports, thymol and carvacrol, which have similarity with cymene in their chemical structures have been shown to be effective on obese animals lipid profiles [31, 35]. With regard to cymene effect, since a decrease is observed in TG and cholesterol levels of treated animals, the hypolipidemic potential of the compound could not be discarded, but further testing with other doses and treatment periods are needed.

Concerning ALT and ALP levels, HFD treated animals showed no significant difference with the control group. Metformin supplementation had also no significant effect on these levels, where cymene caused a significant decrease of ALT and ALP. In reported studies, metformin (1–1.5 g/day) has been effective in reduction of ALT levels [37, 38]. The lack of metformin effect observed in this study may be related to the limited period of the study.

## Conclusions

Use of the simple compound *p*-cymene has shown a significant effect on blood glucose levels of HFD-treated animals, as well as on ALT and ALP levels. Slight effects were also detected toward lipid profiles which may be indicative of a potential of this compound in this regard. Overall, the effects of cymene were comparable with metformin, and show its potential as an alternative to the latter. Further studies involving longer periods are needed to fully validate these results.
